# THE CONTINUOUS LEARNING NEEDS OF PERSONAL SUPPORT WORKERS WHO CARE FOR PEOPLE LIVING WITH DEMENTIA IN LONG-TERM CARE

**DOI:** 10.1093/geroni/igae098.3084

**Published:** 2024-12-31

**Authors:** Grace Norris, Marie Savundranayagam, Afshin Vafaei, Gail Teachman

**Affiliations:** Western University, London, Ontario, Canada; Western University, London, Ontario, Canada; Western University, London, Ontario, Canada; Western University, London, Ontario, Canada

## Abstract

Personal support workers (PSWs) in long-term care (LTC) homes comprise over half of the workforce responsible for providing care to people living with dementia. Compared to other healthcare professionals, PSWs receive the least education, which fails to equip them with the necessary competencies for quality dementia care. To provide optimal care to people living with dementia, PSWs need to be offered opportunities for continuous education that addresses their specific learning needs. Therefore, this study identified and examined the dementia-specific learning needs of PSWs in LTC. Interpretive description guided the secondary qualitative analysis of 22 focus groups with ‘mid-career’ PSWs (n = 39) in LTC. The analysis identified specific learning needs along with ways in which those needs are best met. The learning needs include: 1) addressing responsive behaviours, 2) person-centered communication and attitudes, 3) delirium, and 4) dementia as a chronic condition. Learning needs were most commonly attributed to limited preparation during formal PSW education and a lack of continuous training opportunities throughout their career. Learning needs are best met through experiential methods involving peer learning, feedback, and evaluation within a supportive environment. For the learning needs to mediate practical outcomes, there needs to be an openness to dementia education and a good teamwork culture within LTC. The data generated from this research study will be essential to developing future continuing education for PSWs, contributing to knowledge about dementia care within LTC settings, and improving the quality of care provided to people living with dementia.

